# An Early Fault Diagnosis Method of Rolling Bearings on the Basis of Adaptive Frequency Window and Sparse Coding Shrinkage

**DOI:** 10.3390/e21060584

**Published:** 2019-06-12

**Authors:** Shuting Wan, Bo Peng

**Affiliations:** Department of Mechanical Engineering, North China Electric Power University, Baoding 071000, China

**Keywords:** rolling bearing, feature extraction, early fault diagnosis, adaptive frequency window, sparse coding shrinkage

## Abstract

Early fault information of rolling bearings is weak and often submerged by background noise, easily leading to misdiagnosis or missed diagnosis. In order to solve this issue, the present paper puts forward a fault diagnosis method on the basis of adaptive frequency window (AFW) and sparse coding shrinkage (SCS). The proposed method is based on the idea of determining the resonance frequency band, extracting the narrowband signal, and envelope demodulating the extracted signal. Firstly, the paper introduces frequency window, which can slip on the frequency axis and extract the frequency band. Secondly, the double time domain feature entropy is proposed to evaluate the strength of periodic components in signal. The location of the optimal frequency window covering the resonance band caused by bearing fault is determined adaptively by this entropy index and the shifting/expanding frequency window. Thirdly, the signal corresponding to the optimal frequency window is reconstructed, and it is further filtered by the sparse coding shrinkage algorithm to highlight the impact feature and reduce the residue noise. Fourthly, the de-noised signal is demodulated by envelope operation, and the corresponding envelope spectrum is calculated. Finally, the bearing failure type can be judged by comparing the frequency corresponding to the spectral lines with larger amplitude in the envelope spectrum and the fault characteristic frequency. Two bearing vibration signals are applied to validate the proposed method. The analysis results illustrate that this method can extract more failure information and highlight the early failure feature. The data files of Case Western Reserve University for different operation conditions are used, and the proposed approach achieves a diagnostic success rate of 83.3%, superior to that of the AFW method, SCS method, and Fast Kurtogram method. The method presented in this paper can be used as a supplement to the early fault diagnosis method of rolling bearings.

## 1. Introduction

Rolling bearings, a mechanical component with compact structure and effective reduction of friction loss, are widely used in transportation, the energy and chemical industry, lifting machinery, and other fields. Rolling bearing failure will cause equipment shutdown, machine damage, and even serious personal injury. The fault monitoring and diagnosis of bearings is a useful way to decrease the loss of maintenance and ensure the safety of equipment operation [1,2].

In the initial stage of bearing failure, the defective size of the contact interface is small, the periodic impact force produced by the component interaction is weak, and the feature information is not obvious. Smith et al. [3] discussed the performance of envelope analysis, cepstrum prewhitening, and the benchmark method in extracting failure information from early bearing fault signals. In addition, vibration transmission attenuation, strong background noise, and the interference of multiple vibration sources increase the difficulty of feature extraction [4,5,6]. Aiming at this problem, Miao et al. [7] improved the variational mode decomposition method so that its parameters can be determined adaptively, Jiao et al. [8] proposed the hierarchical discriminating sparse coding method, while Hou et al. [9] put forward an integrated method on the basis of globally optimized sparse coding and approximate SVD. Zheng et al. [10] proposed an NLM-KSVD method for early fault diagnosis of rolling bearings based on shift-invariant K-singular value decomposition (K-SVD) with nonlocal means (NLM) sensitive atom enhancement. Although the above methods can extract weak faults of rolling bearings, complex prior knowledge is needed.

The periodic impact force produced by rolling bearing defects is very weak in the time domain, while the energy distribution is very wide in the frequency domain, arousing the high-frequency resonance of single or multiple mechanical structures such as bearing elements, bearing blocks, etc. The frequency band of resonance response contains abundant fault information, and the fault characteristics can be obtained by envelope demodulation analysis of its corresponding signal. Using resonance demodulation to extract impact response characteristics from the vibration signal is a fast and simple approach for rolling bearing fault diagnosis, the pivotal step of which is to precisely identify the resonance frequency band that contains plentiful fault information. The traditional resonance demodulation approach has three disadvantages. The first one is that the evaluation index cannot distinguish the random impact and periodic impact of the signal, which easily leads to identifying the erroneous resonance band. The second one is that the optimal frequency band may not include the whole resonant frequency band region. The third one is that the parameters of the bandpass filter need to be preset manually. Aiming at these problems, spectral kurtosis was put forward by Dyer [11] originally, then systematically defined by Antoni et al. [12,13,14], and successfully applied to the determination of the resonance frequency band of rolling bearings. In Reference [14], Fast Kurtogram (FK), a fast algorithm based on a finite impulse response filter and short-time Fourier transform, was used to adaptively identify the optimal resonance frequency band. However, the FK algorithm has two obvious shortcomings; one is that the frequency band is decomposed by dichotomy/trichotomy, causing the over-decomposition of the determined resonance band. The other is that the kurtosis index is susceptible to random noise and accidental shock; as a result, the determined frequency band is not the resonance frequency band aroused by the bearing defect. In order to improve the FK algorithm, Lei et al. [15], Gu et al. [16], and Wan et al. [17] proposed improved methods using wavelet packet to construct the filter that better matches the fault impact. Although such improvements can improve the identification accuracy, the resonance frequency band is easily segmented by the adjacent subset because of the pyramid frequency segmentation method. Su et al. [18], Chen et al. [19], and Wan et al. [20] utilized the genetic algorithm, particle swarm optimization algorithm, and water cycle algorithm to determine the function of the Morlet wavelet and asymmetric real Laplace wavelet, and constructed the bandpass filter with an arbitrary Q quality factor to realize adaptive matching of the resonance frequency band. However, such improvements require a lot of prior knowledge, as the optimization algorithms are relatively complex. Wang et al. [21] put forward an adaptive spectral kurtosis method using the maximum spectral kurtosis, continuously expanding the window function in the overall frequency range to determine the optimal resonance frequency band. This improvement can effectively preserve the whole resonance frequency band without the complex optimization process. In addition to the improvement of filter design, many scholars also discussed the evaluation criteria of bandpass filter signal. Yu et al. [22] and Obuchowski et al. [23] described the bearing fault response more accurately by introducing some new statistical models, such as alpha-stable, Jarque–Bera, Kolmogorov–Smirnov, etc. Considering the periodicity of fault impact, Zhang et al. [24] used correlation kurtosis instead of kurtosis to identify the resonance frequency band in diagnosis. Wang et al. [25,26] concluded that the envelope spectrum of the demodulated signal corresponding to the resonance frequency band is sparse, and proposed the Sparsogram method that calculates the kurtosis of the envelope spectrum. Antoni et al. [27] utilized the spectral negentropy of the time domain to represent the impact characteristic, and the spectral negentropy of the frequency domain to represent the cyclostationality characteristic. A method named Infogram based on average spectral negentropy was proposed.

Although most of the background noise in the signal can be filtered by extracting the resonance band caused by bearing fault through a bandpass filter, the noise in the filter passband cannot be filtered out. Especially in the analysis of strong noise signal, the effective removal of noise in the signal can highlight the relevant feature information and increase the failure identification accuracy. Sparse code shrinkage (SCS) was put forward on the basis of maximum-likelihood estimation theory [28]. This method was successfully applied not only to sparse analysis and image de-noising, but also to fault signal diagnosis of mechanical equipment. Lin et al. [29] utilized the maximum-likelihood estimation function of SCS as the soft threshold function of wavelet threshold de-noising for diagnosis pf gear faults, but the diagnosis effort was not ideal under the strong noise interference due to a lack of pre-filtering. Wang et al. [30] put forward an integrated method based on wavelet packet and SCS, which can filter the noise contained in the crack signal, but which needs manual selection of the wavelet packet coefficients to reconstruct the signal for subsequent SCS processing. Yu et al. [31] combined intrinsic time-scale decomposition (ITD) and SCS to diagnose the bearing failure type. However, the baseline of ITD is obtained by the linear transformation of the signal itself, making the waveform of the decomposed proper rotation components appear blurred and distorted, then leading to the inaccurate decomposition of a multi-component AM/FM signal.

Inspired by the above references, a fault diagnosis method on the basis of adaptive frequency window and sparse code shrinkage (i.e., AFW-SCS) is put forward to address the fact that the feature information of rolling bearing early faults is easily submerged by background noise. The AFW can slip along the whole frequency axis and extract the frequency band arbitrarily. Dual-time domain feature entropy is proposed to evaluate the strength of periodic components of the reconstructed signal corresponding to the AFW. According to this evaluation index, the optimal frequency window position is determined adaptively by shifting and expanding the window bandwidth, and covering the resonance band caused by the bearing fault. The reconstructed signal corresponding to the optimal frequency window is further de-noised by the SCS algorithm to make the impact characteristic more prominent and the diagnosis result more accurate. Two experimental signals and the data files of Case Western Reserve University for different operation conditions are applied to validate the effectiveness and the universality of AFW-SCS approach.

The paper consists of five sections. Section 2 illustrates the concept of the frequency window and its adaptive optimization process. Section 3 reviews the theoretical knowledge of SCS. Section 4 exhibits the implementation procedure of the AFW-SCS method. Section 5 validates the feasibility and superiority of the AFW-SCS method through two experimental signals and some existing analysis methods. Section 6 draws some conclusions.

## 2. Adaptive Frequency Window

According to the idea of Reference [32], the adaptive frequency window is proposed to overcome the shortcomings of spectral boundary division caused by frequency domain extreme points. In the next section, the concept of frequency window is firstly introduced. Then, the dual-time domain feature entropy is proposed and used as the evaluation index of the frequency window. Finally, the optimization process of the adaptive frequency window is introduced.

### 2.1. Concept of Frequency Window

**Figure 1:**
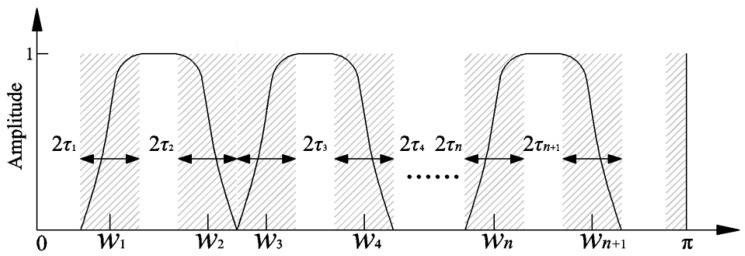
The partitioning of the frequency domain.

Empirical wavelet transform (EWT) can extract the single AM/FM component with a compact spectrum by dividing the Fourier spectrum of the signal and performing orthogonal empirical wavelet transform. Assuming that the signal *x*(*t*) is composed of *N* single AM/FM components, the spectrum range is normalized to [0, π]. In order to extract all single components, it is necessary to divide [0, π] into *N* continuous intervals, and the divided spectrum is as shown in Figure 1. The *N* continuous intervals of the spectrum are expressed as Λ_n_ = [*w_n_*_−1_, *w_n_*] (*n* = 1, 2, ..., *N*). The shadow part with the center of *w_n_* and the width of 2*τ_n_* is the transitional region of each segmentation interval, and satisfies the following requirement:

(1) $$\overset{N}{\underset{n=1}{U}}\Lambda_{n}=\left[0,\pi \right]$$

**Figure 2:**
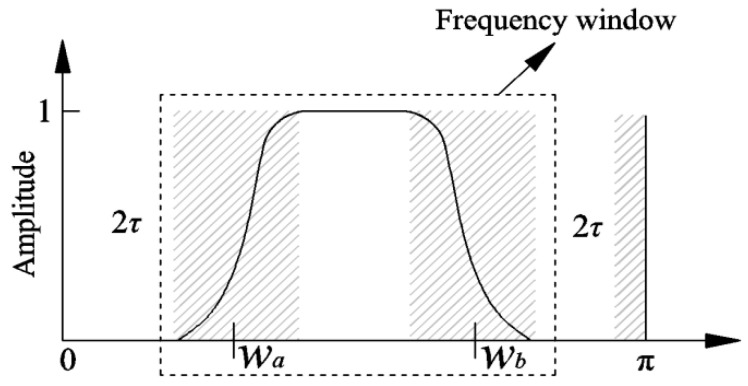
The schematic diagram of the frequency window.

The distribution of extreme points in the signal frequency domain X(f) directly determines the process of signal spectrum segmentation. The extreme points caused by strong background noise will seriously affect the segmentation process, and then affect the EWT processing effect. For this reason, Deng et al. [33] introduced the concept of the frequency window and the signal spectrum segmentation process, which is described in Figure 2. 

As seen, the frequency window Λ is expressed as [*w_a_*, *w_b_*], which represents the center frequency of the upper and lower cut-off bands of the window, where the shadow part is the transitional region of the segment with a width of 2*τ*.

After the signal spectrum is divided by the frequency window, the empirical wavelet function $\hat{\psi }(w)$ is constructed according to the Meyer wavelet construction method, the equation of which is as follows:

(2) $$\hat{\psi }(w)=\left\{ \begin{array}{ll} 1 & ,\;w_{a}+\tau \leq \left|w\right|\leq w_{b}-\tau \\ \cos\left[\frac{\pi }{2}\beta \left(\frac{1}{2\tau }\left(\left|w\right|-w_{b}+\tau \right)\right)\right] & ,w_{b}-\tau \leq \left|w\right|\leq w_{b}+\tau \\ \sin\left[\frac{\pi }{2}\beta \left(\frac{1}{2\tau }\left(\left|w\right|-w_{a}+\tau \right)\right)\right] & ,w_{a}-\tau \leq \left|w\right|\leq w_{a}+\tau \\ 0 & ,\mathrm{else} \end{array}\right.$$

where the relevant parameters satisfy the following requirement:

(3) $$\left\{ \begin{array}{l} \beta \left(x\right)=x^{4}(35-84x+70x^{2}-20x^{3})\\ \tau =\gamma w_{a}\\ \gamma <\left(w_{b}-w_{a}\right)/\left(w_{b}+w_{a}\right) \end{array}\right..$$

The wavelet coefficient can be calculated as

(4) $$W_{ff}(t)=\langle x(t),\psi (t)\rangle =\int x(\tau )\psi (\tau -t)d\tau =F^{-1}\left[x(w)\hat{\psi }(w)\right],$$

where 〈•〉 represents the inner product, and $F^{-1}\left(•\right)$ represents the inverse Fourier transform.

The signal x^*^(*t*) corresponding to the frequency window can be reconstructed as follows:

(5) $$x^{*}(t)=W_{ff}(t)*\psi (w)=F^{-1}\left[{\hat{W}}_{ff}(w)\hat{\psi }(w)\right],$$

where ∗ represents the convolution operation, and ${\hat{W}}_{ff}(w)$ is the Fourier transform of $W_{ff}(t)$.

The defect of rolling bearing parts can cause resonance, and the resonance band contains abundant fault information. If the frequency domain window covers the resonance band, the signal reconstructed based on this frequency window has obvious fault characteristics. The frequency window parameters *w_a_* and *w_b_* can slide freely, and the bandwidth of the frequency window is variable. How to determine the frequency window that covers the whole bearing fault resonance frequency band without manually doing so is a challenge.

### 2.2. Dual-Time Domain Feature Entropy

Dual-time domain transformation (DTDT) is an analysis method on the basis of generalized S-transform (GST) [34] and Fourier transform, representing the non-stationary feature in the dual-time domain. The GST is an extension of S-transform (ST). The ST of one-dimensional signal *x*(*t*) is illustrated as follows [35]:

(6) $$ST_{x}(t,f)=\int_{-\infty }^{+\infty }x(\tau )w(\tau -t,\sigma (f))\exp\left(j2\pi f\tau \right)d\tau ,$$

where *τ* represents the time-shift factor, *f* represents the carrier frequency, and *w*(*τ − t*) represents the Gauss window, the standard deviation of which is *σ*(*f*) = 1/∣*f*∣. The width of the window function (WF) decreases with the increase of carrier frequency. In order to enhance the adaptability of WF of ST, two adjusting factors *k* and *p* are introduced into thw standard deviation *σ*(*f*).

(7) $$\sigma (f)=k/{\left|f\right|}^{p}.$$

The window function is rewritten as

(8) $$w(\tau -t)=\frac{{\left|f\right|}^{p}}{k\sqrt{2\pi }}\exp\left[-\frac{f^{2p}{\left(\tau -t\right)}^{2}}{2k^{2}}\right].$$

The GST of one-dimensional signal *x*(*t*) can be illustrated as follows [12]:

(9) $${\mathrm{GST}}_{x}(\tau ,f)=\frac{{\left|f\right|}^{p}}{k\sqrt{2\pi }}\int_{-\infty }^{+\infty }x(\tau )\exp\left[-\frac{f^{2p}{\left(\tau -t\right)}^{2}}{2k^{2}}-2\pi f\tau \right]d\tau .$$

The essence of GST is to replace the fixed window function of short-time Fourier transform with the Gaussian window function, the standard deviation of which varies according to Equation (7). GST has a high time resolution for high-frequency signals and a high frequency resolution for low-frequency signals, showing a good time–frequency aggregation performance. When *k* = 1 and *p* = 1, the GST degenerates to ST.

GST can map one-dimensional time signals to the two-dimensional time–frequency plane, and Fourier inverse transform can transform the frequency domain to the time domain. Therefore, DTDT can be obtained by inverse Fourier transform of the results of GST at different times, the mathematical equation of which is described as

(10) $$\begin{array}{ll} DTDT_{x}(\tau_{1},\tau_{2}) & =\int_{-\infty }^{+\infty }{\mathrm{GST}}_{x}(\tau_{1},f)\exp\left[2\pi f\tau_{2}\right]df\\ & =\int_{-\infty }^{+\infty }\int_{-\infty }^{+\infty }x(t)w(\tau_{1}-t)\exp\left[2\pi f(\tau_{2}-t)\right]dtdf \end{array}.$$

Because the vibration signal *x* = {*x*(*kT*), *k* = 0, 1, 2, …, *N* − 1} (*N* represents the signal length and *T* represents the sample interval) collected by sensors is a discrete signal, the DTDT should be discretized. Using Fourier transform and convolution theory, Equation (4) can be rewritten as

(11) $${\mathrm{GST}}_{x}(\tau ,f)=\int_{-\infty }^{+\infty }X(\alpha +f)\exp\left[-\frac{2\pi^{2}\alpha^{2}k^{2}}{f^{2p}}+i2\pi \alpha \tau \right]d\alpha ,$$

where *X*(·) is the Fourier transform of *x*(*t*), and *α* represents the shifting frequency. Setting f→n/(NT) and τ→kT, the discrete generalized S-transform can be expressed as

(12) $${\mathrm{GST}}_{x}(kT,\frac{n}{NT})=\sum_{m=0}^{N-1}X\left[\frac{n+m}{NT}\right]\exp\left[-\frac{2n^{2}k^{2}m^{2}}{n^{2p}}+i\frac{2\pi mj}{N}\right],$$

where *k, m, n* = 0, 1, 2, …, *N* − 1.

Similarly, the discrete expression of DTDT can be deduced as follows:

(13) $$DTDT_{x}(kT,iT)=\sum_{n=0}^{N-1}{\mathrm{GST}}_{x}(kT,\frac{n}{NT})\exp\left[2\pi nk\right],$$

where *i, k, n* = 0, 1, 2, …, *N* − 1.

After DTDT is performed on the signal, an *N* × *N* dimension complex matrix, named the dual-time domain transformation matrix (DTDTM), is obtained. The signal energy is mainly concentrated near the diagonal position of DTDTM; the low-frequency component of the signal will produce larger energy leakage, while the high-frequency component will not produce obvious energy leakage. In other words, at the diagonal position of DTDTM, the high-frequency component can obtain higher gain than the low-frequency component. According to this characteristic, the diagonal elements of DTDTM are extracted to construct a new one-dimensional signal, which can reflect the high-frequency characteristics with lower amplitude of the original signals.

With the application of entropy theory in various fields, various forms of entropy gradually evolved, such as information entropy, sample entropy, approximate entropy, etc. This present paper puts forward a new entropy method named dual-time domain feature entropy (DTDFE), which consists of information entropy and DTDT. According to the definition of information entropy, the equation for calculating DTDFE can be constructed as follows:

(14) $$\begin{array}{l} DTDFE=-\left[\sum_{i=1}^{N}P_{i}\ln(P_{i})\right]\ln(N)\\ P_{i}=A(i)/\sum_{i=1}^{N}A(i)\\ \sum_{i=1}^{N}P_{i}=1 \end{array}\},$$

where *A*(*i*) is the amplitude of the one-dimensional signal constructed by the diagonal elements of DTDTM. DTDFE can not only make full use of the information obtained by the dual-time domain analysis and better characterize the dynamic changes of the signal, but it can also suppress the low-frequency clutter interference.

**Figure 3:**
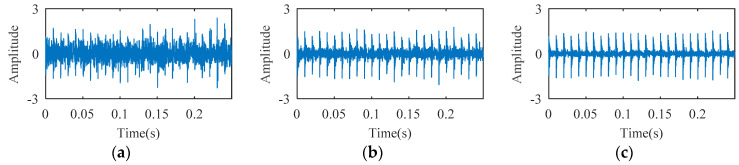
Temporal waveform of simulation fault signal under different signal-to-noise ratios (SNRs): (**a**) 3 dB; (**b**) 6 dB; (**c**) 10 dB.

**Figure 4:**
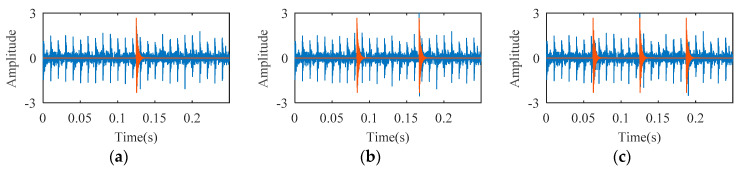
Temporal waveform of 6-dB simulation fault signal under different random impacts: (**a**) one random impact (*t*); (**b**) two random impacts; (**c**) three random impacts.

**Figure 5:**
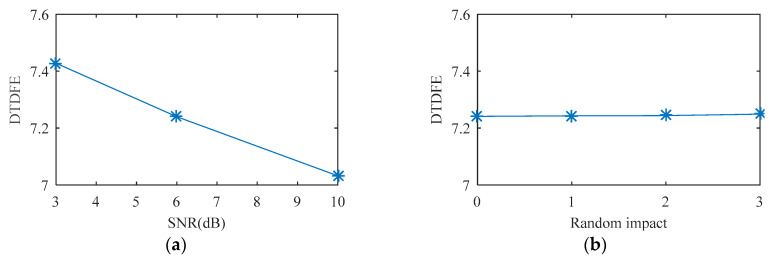
Variation tendency of dual-time domain feature entropy (DTDFE) with (**a**) SNR, and (**b**) random impact.

Rolling bearing fault simulation signals are obtained using periodic impulse signals superimposed with Gaussian noise signals. The temporal waveforms of bearing fault simulation signals with signal-to-noise ratios (SNR) of 3 dB, 6 dB, and 10 dB are described in Figure 3. As shown in Figure 4, one, two, and three random impacts are added to the 6-dB fault simulation signal described in Figure 3b. The red and blue line waveforms represent the random impact and the 6-dB fault simulation signal, respectively. The value of DTDFE of the signals shown in Figure 3 are calculated, the variation tendency of which is shown in Figure 5a. As seen, DTDFE decreases with the increase of periodic pulse intensity in the signal. The value of DTDFE of the signals shown in Figure 4 are calculated, the variation tendency of which is shown in Figure 5b. As seen, DTDFE changes little with different random impacts. In summary, DTDFE can not only evaluate the strength of periodic components in signal, but also immunize them against random impact, with better robustness.

### 2.3. Optimization Process of Adaptive Frequency Window

The defect of rolling bearing parts can arouse resonance, and the resonance band contains abundant fault information. If the frequency domain window covers the resonance band, the analysis of the constructed signal corresponding to the resonance band will reduce the difficulty and improve the accuracy of diagnosis. The resonance band can be determined adaptively by shifting and expanding the frequency window, the specific steps of which are as follows:

Step 1: The fault frequency *F_f_* of the diagnostic object is calculated, and the frequency band of the initial reference frequency window (RFW) is set as [*w_a_*, *w_b_*], *w_a_* = 0 and *w_b_* = *w_f_* = 3 × *F_f_*. *w_f_* represents the initial bandwidth of the frequency window, the value of which is set according to the conclusion that the optimal fault bandwidth should not be less than three times the fault feature frequency [25,26].

Step 2: The shifting frequency window (SFW) is obtained by moving the RFW along the frequency axis, the frequency band of which is set as [*w_b_*, *w_b_* + *w_f_*]. The expanding frequency window (EFW) consists of RFW and SFW, the frequency band of which is set as [*w_a_*, *w_b_* + *w_f_*]. The DTDFE of the narrowband signals corresponding to RFW, EFW, and SFW is calculated, the values of which are recorded as FE1, FE2, and FE3, respectively. 

Step 3: The values of FE1, FE2, and FE3 determine the shifting and expanding of the frequency domain window. The relationship of the above entropy values is judged by Equation (15).

(15) FE 2≤min(FE   1,FE   3).

If the condition is satisfied, the narrowband signal corresponding to EFW contains more fault information and, thus, is conducive to fault diagnosis. The upper and lower cut-off frequency of EFW is used as that of the new RFW, and the new SFW and EFW are also constructed according to Step 2. The entropy values of the new RFW, EFW, and SFW are calculated, the relationships of which are judged by Equation (15). If the condition is not satisfied, the narrowband signal corresponding to RFW or SFW has the higher SNR and, thus, is conducive to fault diagnosis. The upper and lower cut-off frequency of SFW is used as that of the new RFW, and the new SFW and EFW are also constructed according to Step 2. The entropy values of the new RFW, SFW, and EFW are calculated, the relationships of which are judged by Equation (15).

Step 4: Step 3 is repeated until the upper limit of the RFW (i.e., *w_b_* + *w_f_*) is greater than *f_s_*/2. The RFW with the minimum value of DTDFE is the optimal frequency window, the extracted frequency band of which covers the whole resonance band aroused by the bearing fault. That is to say, the reconstructed signal of the optimal frequency window has the most fault information.

**Figure 6:**
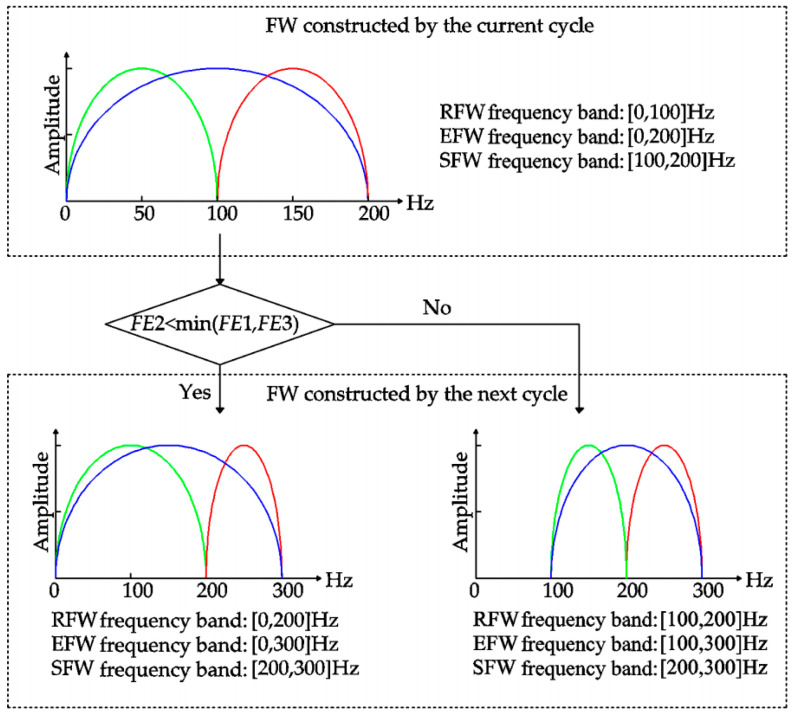
The effect of DTDFE criteria on the frequency window (FW).

In order to clearly describe the influence of DTDFE criterion on RFW, SFW, and EFW, a schematic diagram is drawn in Figure 6. The frequency band of the initial RFW is [0, 100] Hz. The green, blue, and red lines correspond to RFE, EFW, and SFW, respectively.

## 3. Sparse Coding Shrinkage

The sparse coding shrinkage algorithm [28] is a de-noising method on the basis of sparse coding theory to deduce the hidden non-Gaussian components by using statistical characteristics of data. Hyvarinen proposed the following mathematical model to represent the probability density function of non-Gaussian signals:

(16) $$p\left(x\right)=\frac{1}{2d}\frac{\left(\alpha +2\right){\left[\alpha \left(\alpha +1\right)/2\right]}^{\left(\alpha /2+1\right)}}{{\left[\sqrt{\alpha \left(\alpha +1\right)/2}+\left|x/d\right|\right]}^{\left(\alpha +3\right)}},$$

where *x* represents the signal with non-Gaussian statistical properties, *d* represents the standard deviation of the signal *x*, and *α* represents the parameter to control the sparsity of the probability density function. The greater the value of *α* is, the sparser the probability density function will be. For non-Gaussian signals with impact characteristics, the value of *α* can be set to 0.1.

The impulse component of the bearing fault vibration signal is a typical non-Gaussian component. Therefore, the algorithm is used to further process the results of AFW, making the impulse feature more obvious and the fault identification more accurate. The vibration signal of fault rolling bearing *y*, represented as Equation (17), consists of the defect impulse signal *x* and the Gauss white noise signal *v*.

(17) y=x+v,

where the statistical characteristics of *x* show non-Gaussian properties. The mean and variance of noise signal *v* are 0 and *σ*^2^, respectively.

According to the sparse probability density function model represented by Equation (13), the estimated signal $\hat{x}$ of the impulse signal *x* can be deduced from the vibration signal *y* by using the maximum-likelihood estimation theory.

(18) $$\beta =\sqrt{\alpha \left(\alpha +1\right)/2},$$

(19) σ=mad(y),

(20) $$d=\sqrt{\sigma_{y}^{2}-\sigma^{2}},$$

(21) $$\hat{x}=\mathrm{sign}(y)\times \max\left\{0,\frac{\left|y\right|-\beta d}{2}+\frac{1}{2}\sqrt{{\left(\left|y\right|+\beta d\right)}^{2}-4\sigma^{2}\left(\alpha +3\right)}\right\},$$

where *σ* represents the standard deviation of noise signal *v*, $\sigma_{y}^{2}$ represents the standard deviation of vibration *y*, and mad represents the mean absolute deviation of the values in the signal.

## 4. Diagnosis Method Based on AFW and SCS

**Figure 7:**
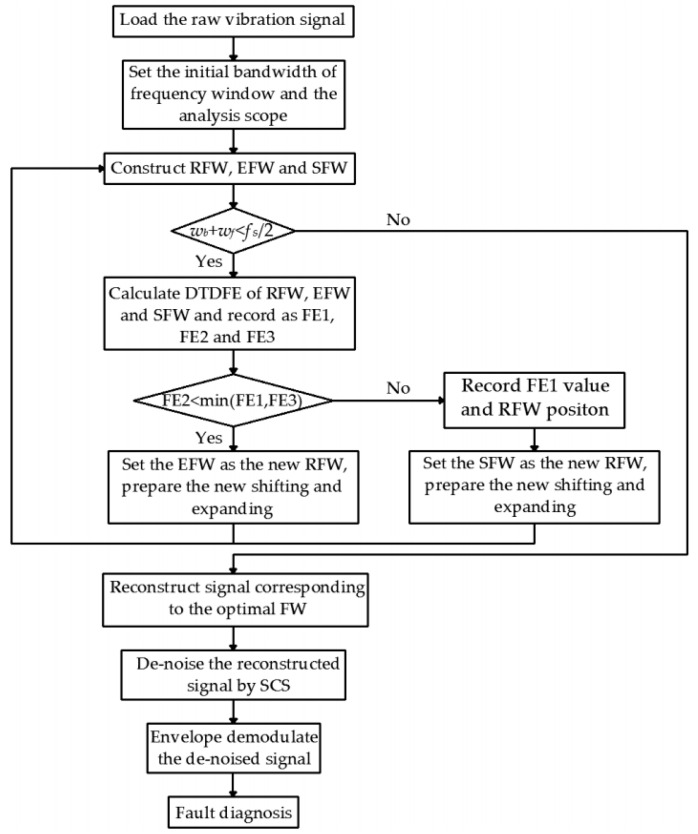
The diagnostic flow chart of the adaptive frequency window with sparse coding shrinkage (AFW-SCS).

AFW can filter the most background noise in the signal by extracting the resonance band caused by bearing faults through a specific bandpass filter. However, the noise in the filter passband cannot be filtered out. The impulse components of bearing faults are usually non-Gaussian, while the noise components are Gaussian. SCS can effectively separate the Gaussian signal and non-Gaussian signal; thus, this algorithm is used to further process the narrowband signal obtained by AFW to highlight the impact component and then effectively eliminate the residual noise. Combining the advantages of AFW and SCS in signal processing, a fault diagnosis method named AFW-SCS is proposed in the present paper to solve the misdiagnosis or missed diagnosis caused by the difficulty of detecting the early fault feature information submerged in background noise. Comparing with the existed diagnosis methods based on resonance demodulation theory, this current work has three improvements. Firstly, the proposed AFW method can avoid the resonance band segmentation, keep the resonance band to the maximum extent, and extract more fault information. The adaptive process of AFW can be implemented by shifting and expanding the frequency window without the help of complex optimization algorithms. Secondly, the proposed DTDFE can effectively evaluate the strength of periodic impact in the signal and is not disturbed by random impact. The width and position of the resonance band can be determined more accurately by using DTDFE as the evaluation index of the frequency window. Thirdly, the SCS method is used to further eliminate non-Gaussian components of the extracted signal, so that the impact components can be highlighted, and the residue noise in the extracted signal can be reduced. The specific diagnostic steps and diagnostic flow chart of AFW-SCS are illustrated in Algorithm 1 and Figure 7, respectively.

**Algorithm 1.** The execution step of AFW-SCS 1: Load original vibration signal, set sampling frequency *f_s_*, and calculate fault feature frequency *F_f_*.2: Set the initial bandwidth of frequency window as *w_f_* = 3 × *F_f_* and the analysis scope as [0, *f_s_*/2].3: Construct RFW, EFW, and SFW. If RFW is [*w_a_*, *w_b_*], EFW is [*w_a_*, *w_b_* + *w_f_*] and SFW is [*w_b_*, *w_b_* + *w_f_*].4: Run the judgment condition, *w_b_* + *w_f_* < *f_s_*/2. If condition is met, run Steps 4, 5, and 6. If condition is not satisfied, run Step 7.5: Calculate DTDFE of RFW, EFW, and SFW, and record as FE1, FE2, and FE3. 6: Run the judgment condition, FE2 < min(FE1, FE3). If conditions are met, set the EFW as the new RFW, and return to Step 3. If condition is not satisfied, record FE1 value and RFW position (i.e., upper and lower cut-off frequency), set the SFW as the new RFW, and return to Step 3. 7: Select the frequency window corresponding to the minimum entropy and reconstruct the signal corresponding to the frequency window. 8: Perform SCS on the reconstructed signal to further reduce the noise.9: Demodulate the de-noised signal by envelope operation, and calculate its envelope spectrum. 10: Diagnose the fault type by contrasting the frequency corresponding to the larger-amplitude spectral line in the envelope spectrum and the failure characteristic frequency.

## 5. Rolling Bearing Fault Signal Analysis Case

In this section, the vibration signals of the ball bearing under outer ring fault and the cylindrical bearing under inner ring fault are utilized to testify the feasibility, generality, and superiority of this diagnosis approach proposed in the present paper. The signal acquisition analyses are completed using the Case Western Reserve University (CWRU) [36] and North China Electric Power University (NCEPU) datasets.

### 5.1. Case 1: Ball Bearing

**Figure 8:**
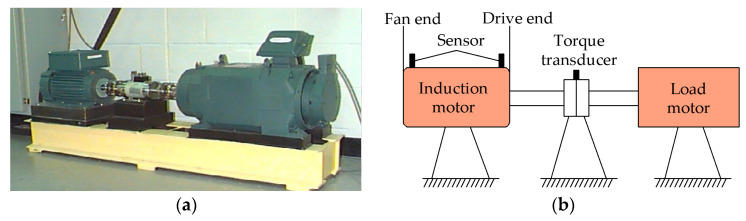
Case Western Reserve University (CWRU) platform: (**a**) photograph; (**b**) structural sketch.

The vibration signals of the SKF6203 ball bearing under outer ring fault are collected from the experimental platform of CWRU. Figure 8a,b describe the photograph and structural sketch of the platform, respectively. Table 1 illustrates the structure parameters of the SKF6203 ball bearing. The acceleration sensors used to acquire the signal are fixed at the fan end and the drive end of the induction motor. In the present paper, the 2048 sample points of the fan-end accelerometer data from the 132nd data file (i.e., OR007@6_3) are selected for analysis. The corresponding defective position, defective diameter, shaft speed, sample frequency, and load are the outer ring, 0.007 inches, 1750 rpm, 12,000 Hz, and 2 horsepower (HP), respectively. The outer ring fault feature frequency *f_o_*, i.e., ballpass frequency of outer ring (BPFO) [37], is 105 Hz, calculated using Equation (22).

(22) $$\mathrm{BPFO}=\frac{Z}{2}(1-\frac{d}{D}\cos\alpha )\frac{n}{60},$$

where *Z*, *d*, *D*, *α*, and *n* represent the roller number, roller diameter, pitch diameter, contact angle, and shaft speed, respectively.

**Figure 9:**
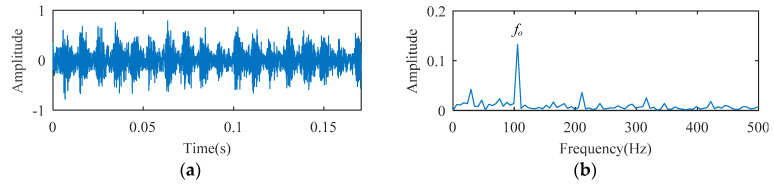
The ball bearing fault experimental signal: (**a**) temporal waveform; (**b**) envelope spectrum.

The temporal waveform and envelope spectrum of the ball bearing fault experimental signal are described in Figure 9. In the envelope spectrum of Figure 9, a spectral line with higher amplitude appears, and its frequency corresponds to 105 Hz (i.e., *f_o_*). The higher harmonic spectral lines corresponding to *f_o_* are not obvious and can easily be neglected. 

**Figure 10:**
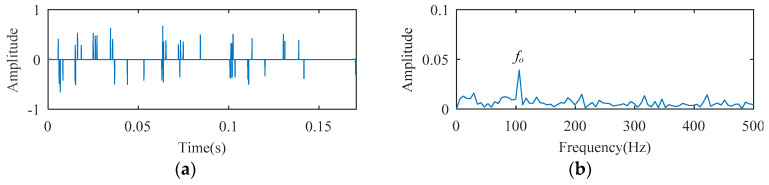
The diagnosis results of SCS: (**a**) temporal waveform; (**b**) envelope spectrum.

**Figure 11:**
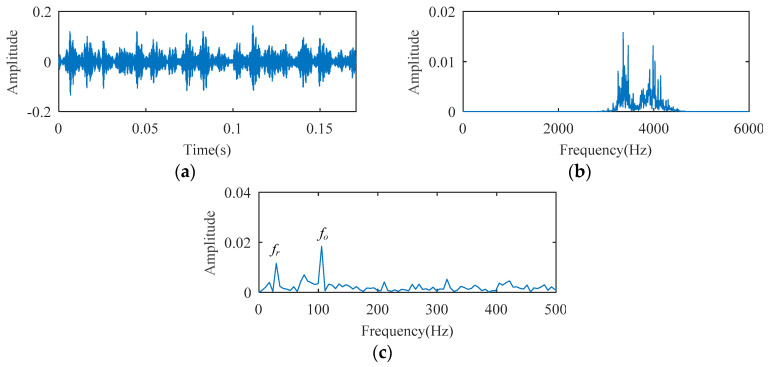
The diagnosis results of AFW: (**a**) extracted frequency band; (**b**) temporal waveform; (**c**) envelope spectrum.

**Figure 12:**
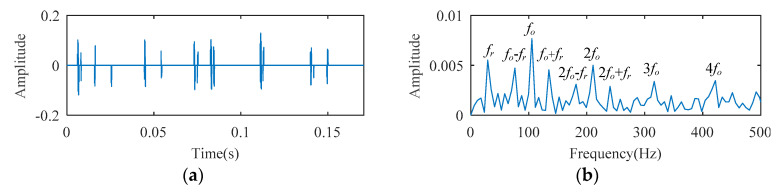
The diagnosis results of AFW-SCS: (**a**) temporal waveform; (**b**) envelope spectrum.

**Figure 13:**
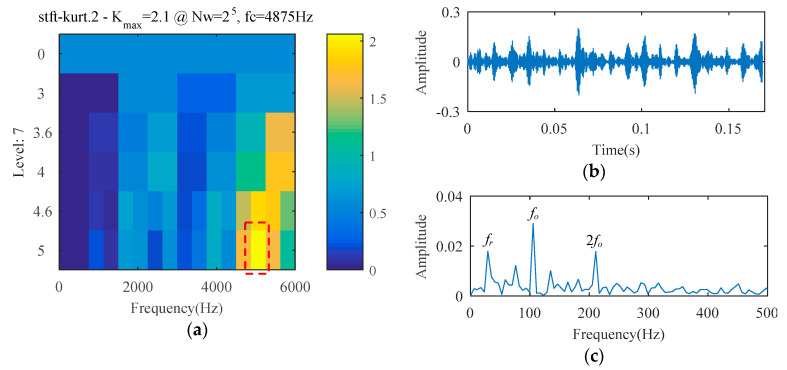
The diagnosis results of the Fast Kurtogram (FK) method: (**a**) kurtogram; (**a**) temporal waveform; (**b**) envelope spectrum.

The SCS method is utilized to analyze the ball bearing fault experimental signal, and the analysis result is illustrated in Figure 10. Although the signal temporal waveform becomes sparse in Figure 10, only a spectral line corresponding to *f_o_* appears in the envelope spectrum of Figure 10. The spectral lines corresponding to the higher harmonics of *f_o_* are absent. Using the AFW method to process this signal, the optimal frequency window is determined adaptively by the shifting and expanding operation, the upper and lower cut-off frequencies of which are 2835 Hz and 4725 Hz. The temporal waveform and fast fourier transform (FFT) spectrum of the reconstructed signal corresponding to the optimal frequency window are shown in Figure 11a,b. The reconstructed signal is demodulated by the envelope operation, and its envelope is obtained as described in Figure 11c. As seen, there are two spectral lines with high amplitude, the frequency of which correspond to 105 Hz (i.e., *f_o_*) and 25 Hz (i.e., *f_r_*, the rotation frequency). The higher harmonic spectral lines corresponding to *f_o_* are still not apparent. The diagnosis results of the SCS and AFW method may lead to misdiagnosis or missed diagnosis. The reconstructed signal obtained by the AFW method is further processed by the SCS method, and the diagnosis results of the AWF-SCS method are described in Figure 12. In the temporal waveform of Figure 12, the signal exhibits more sparsity than that of Figure 10a. In the envelope spectrum of Figure 12, the spectral lines corresponding to the fault frequency *f_o_* and its higher harmonic 2*f_o_*, 3*f_o_*, and 4*f_o_* can be clearly seen. In addition, the phenomenon of rotation frequency modulation is obvious, and the spectral lines corresponding to the sidebands of *f_o_* and 2*f_o_* (i.e., *f_o_* ± *f_r_*, 2*f_o_* ± *f_r_*) are also quite apparent. According to these results, the fault type can be definitively identified as an outer ring fault. For comparison, the Fast Kurtogram (FK) method [14] is used to process this ball bearing experimental signal. In the kurtogram of Figure 13, the resonance band determined by FK is marked by a red line, and the temporal waveform and envelope spectrum of its corresponding signal are illustrated in Figure 13b,c. As seen, the spectral lines corresponding to *f_r_*, *f_o_*, and 2*f_o_* can be seen.

In this case, comparing the diagnosis results of SCS, AFW, FK, and AFW-SCS, the proposed method can extract more fault information and, thus, is conducive to accurately diagnosing the fault type as an outer ring fault.

### 5.2. Case 2: Cylindrical Bearing

**Figure 14:**
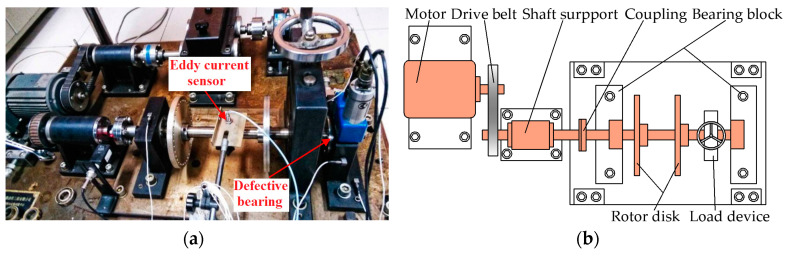
North China Electric Power University (NCEPU) platform: (**a**) photograph; (**b**) sensor position; (**c**) structural sketch.

**Figure 15:**
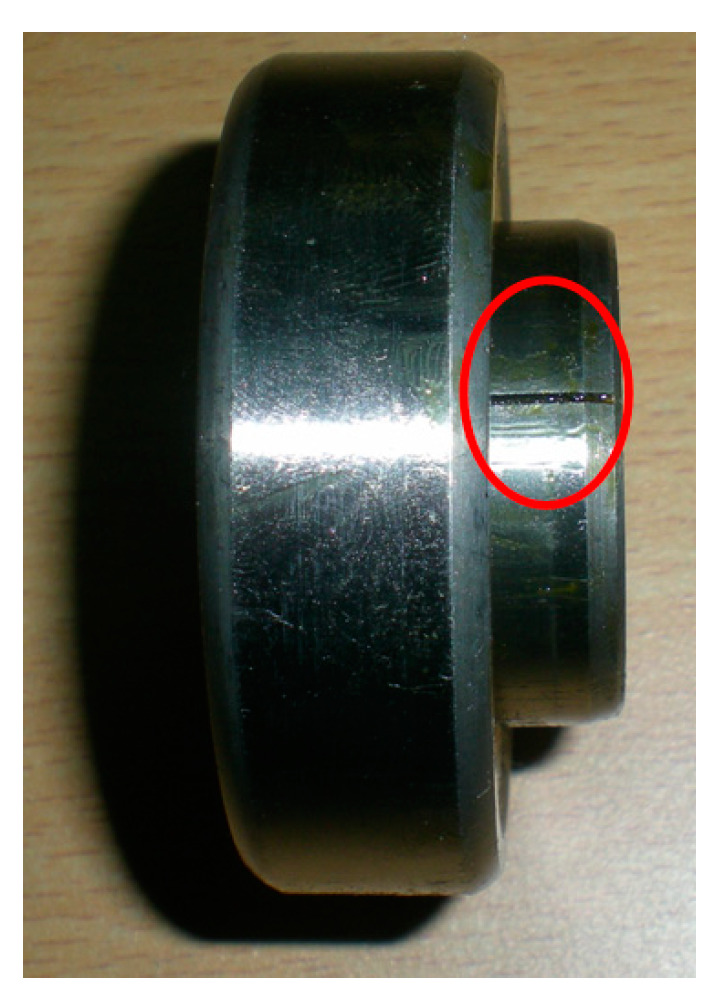
Physical photo of the N205 cylindrical bearing with outer ring defect.

The vibration signals of the N205 cylindrical bearing under inner ring fault are collected on the QPZZ-II fault simulation platform for rotating machinery of the NCEPU. An eddy current sensor is used to acquire data. Figure 14a,b show the physical photo and the structural sketch of the QPZZ-II platform, respectively. The defective bearing is installed at the right bearing block. Table 2 illustrates the structure parameters of the N205 cylindrical bearing. To obtain the bearing vibration signal under an inner ring fault running station, a defect with a width of 0.2 mm and depth of 0.1 mm on the inner ring surface is machined using electric discharge machining technology, the physical photo of which is shown in Figure 15. Throughout the entire process of the experiment, the shaft speed, the sampling frequency, and load are set as 1440 rpm, 12,800 Hz, and 0 HP, respectively. The inner ring fault feature frequency *f_i_*, i.e., ballpass frequency of inner ring (BPFI) [37], is 172 Hz, calculated using Equation (23).

(23) $$\mathrm{BPFI}=\frac{Z}{2}(1+\frac{d}{D}\cos\alpha )\frac{n}{60},$$

where the definitions of *Z*, *d*, *D*, *α*, and *n* are the same as those in Equation (23). 

**Figure 16:**
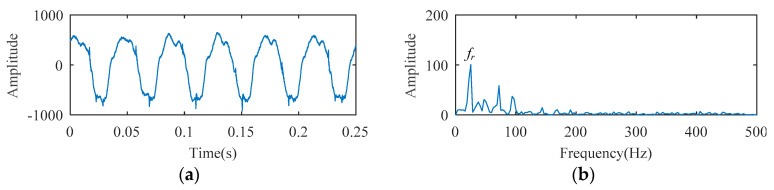
The cylindrical bearing fault experimental signal: (**a**) temporal waveform; (**b**) envelope spectrum.

The temporal waveform and envelope spectrum of the cylindrical bearing fault experimental signal are described in Figure 16. As shown in the temporal waveform of Figure 16, six similar local waveforms appear in 0.25 s. This signal can be considered as a harmonic signal, the period of which is 0.041 s (0.25/6 ≈ 0.041). In the envelope spectrum of Figure 16, the spectral line corresponding to 24 Hz (i.e., *f_r_*, the rotation frequency) can be clearly seen, but the fault frequency cannot be detected. Fault characteristics are submerged by the rotation frequency carrier signal. 

**Figure 17:**
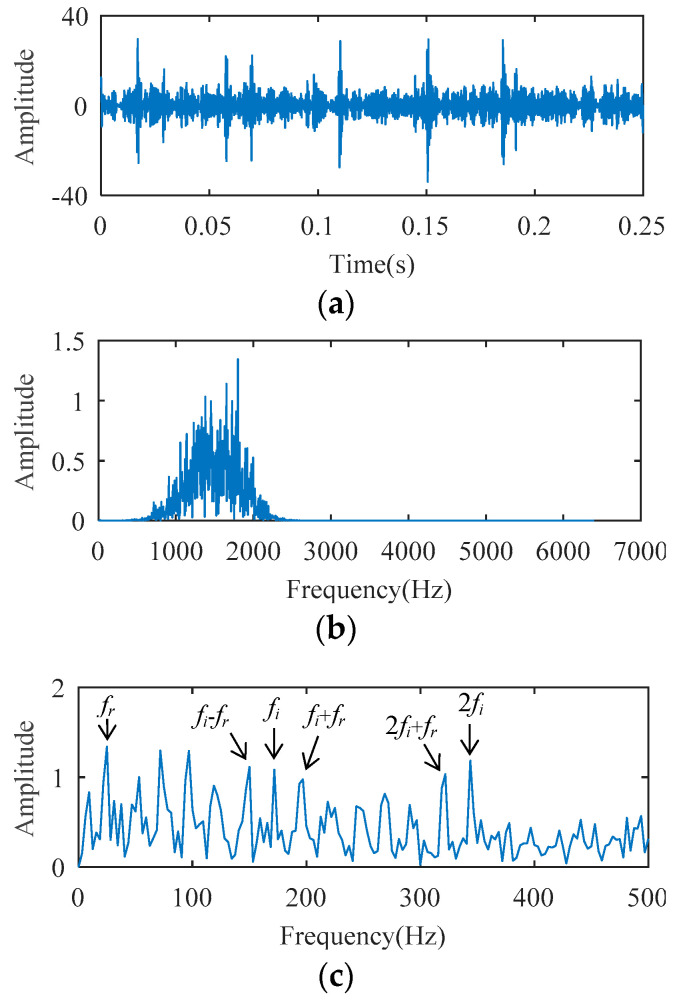
The diagnosis results of AFW: (**a**) extracted frequency band; (**b**) temporal waveform; (**c**) envelope spectrum.

**Figure 18:**
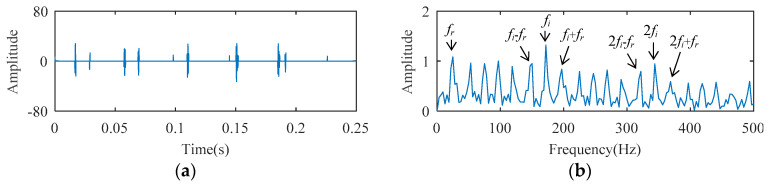
The diagnosis results of AFW-SCS: (**a**) temporal waveform; (**b**) envelope spectrum.

**Figure 19:**
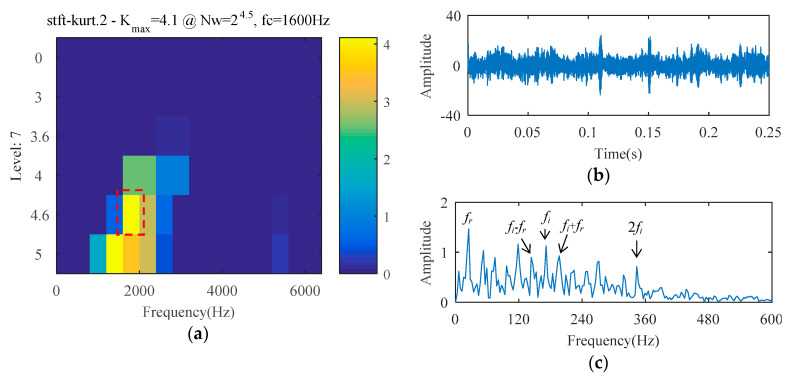
The diagnosis results of FK: (**a**) kurtogram; (**b**) temporal waveform; (**c**) envelope spectrum.

For the fact that the SCS method cannot analyze the harmonic signal, it is impossible to process this bearing signal to realize fault diagnosis. The AFW method is used to analyze the signal, and the upper and lower cut-off frequencies of the optimal frequency window are 516 Hz and 2064 Hz. The temporal waveform and FFT spectrum of the reconstructed signal corresponding to the optimal frequency window are described in Figure 17. As shown in the temporal waveform of Figure 17, the impact characteristics appear. Figure 17c is the envelope spectrum of the reconstructed signal, where the spectral lines corresponding to 24 Hz (i.e., *f_r_*), 172 Hz (i.e., *f_i_*), and 344 Hz (i.e., 2*f_i_*) can be detected, but the amplitude of those spectral lines are not prominent. The spectral line amplitude corresponding to the sideband *f_o_* − *f_r_* is greater than that of *f_o_*, and the spectral line corresponding to the sideband 2*f_o_* + *f_r_* is absent. These results may lead to misdiagnosis or missed diagnosis. The reconstructed signal obtained by the AFW method is further processed by the SCS method, and the diagnosis results of the AWF-SCS method are described in Figure 18. In the temporal waveform of Figure 18, the impact characteristics of the signal are more obvious and there is no noise interference. In the envelope spectrum of Figure 18, the spectral line amplitudes corresponding to 24 Hz, 172 Hz, and 344 Hz are more prominent than those of Figure 17c. In addition, the spectral lines corresponding to the sidebands of *f_o_* and 2*f_o_* (i.e., *f_o_* ± *f_r_*, 2*f_o_* ± *f_r_*) are more explicit than those of Figure 17c. According to these results, the fault type can be definitively identified as inner ring fault. For comparison, the Fast Kurtogram (FK) method [9] is used to process this cylindrical bearing experimental signal. In the kurtogram of Figure 19, the resonance band determined by FK is marked by the red line, and the temporal waveform and envelope spectrum of its corresponding signal are described in Figure 19b,c. As seen, the spectral lines corresponding to *f_r_*, *f_i_*, 2*f_i_*, and *f_o_* ± *f_r_* can be seen, but the amplitude of those spectral lines is less than that of the interference frequency. 

In this case, comparing the diagnosis results of SCS, AFW, FSK, and AFW-SCS, the proposed method can extract more fault information and, thus, is conducive to accurately diagnosing the fault type as an inner ring fault.

### 5.3. Further Discussion

In order to evaluate the universality of the proposed method under different operation conditions, the 105th, 119th, 132nd, 170th, 186th, 199th, 209th, 222nd, 236th, 108th, 188th, and 237th data files of CWRU are selected for analysis. The data length, sample frequency, and acquisition location of these selected signals are as follows: 2048 sample points, 12,000 Hz, and drive end; the corresponding operation conditions of these signals, such as defect position (DP), defect diameter (DD), and motor load (ML), are listed in Table 3. The AFW-SCS, AFW, SCS, and FK methods are utilized to analyze these signals. If the fault characteristic frequency and its second- and third-order harmonics can be extracted clearly, the diagnosis is successful. Otherwise, the diagnosis is a failure. The diagnosis results of these methods are summarized in Table 4. The diagnostic success rates of the AFW-SCS, AFW, SCS, and FK methods are 83.3% (10/12), 41.7% (5/12), 16.7% (2/12), and 33.3% (4/12), respectively, indicating that the AFW-SCS method is effective in extracting more failure information, achieving early fault diagnosis of the rolling bearing, and having higher reliability.

## 6. Conclusions

This present paper put forward an adaptive approach, named AFW-SCS, for the diagnosis of rolling bearing weak faults. The proposed method is based on the idea of determining the resonance frequency band, extracting the narrowband signal, and envelope demodulating the extracted signal. The diagnosis results of the bearing vibration signals of CRWU and NCEPU illustrate that the proposed AFW-SCS method can extract more failure information, highlight the early failure feature, and accurately diagnose the bearing failure type. Compared with the published approach, the AFW-SCS method has three highlights. Firstly, the AFW method can avoid the resonance band segmentation, keep the resonance band to the maximum extent, and extract more fault information. The adaptive process of AFW can be implemented by shifting and expanding the frequency window without the help of complex optimization algorithms. Secondly, DTDFE can effectively evaluate the strength of periodic impact in the signal and is not disturbed by random impact. The width and position of the resonance band can be determined more accurately using DTDFE as the evaluation index of the frequency window. Thirdly, the SCS method can further eliminate non-Gaussian components of the signal extracted by the AFW method, so that the impact characteristics in the extracted signal can be highlighted, the residue noise in the extracted signal can be reduced, and the fault diagnosis accuracy can be improved.

Although the AFW-SCS method based on resonance demulation has advantages in the determination of resonance frequency band and the enhancement of fault feature information, its diagnostic success rate under strong noise interference is not ideal. Diagnostic methods based on dictionary learning, such as the NLM-KSVD method, perform better in anti-strong noise interference, but the process of acquiring dictionaries is complex and difficult to understand. Comparatively speaking, the AFW-SCS method is easier to understand and requires less prior knowledge. In any case, the AFW-SCS method and the NLM-KSVD method are complements to their respective algorithmic domains. In future research, we will systematically analyze the application scope of the AFW-SCS method, thoroughly study how to simplify the process of acquiring dictionaries in the NLM-KSVD method, try to combine the advantages of the two methods, and propose a bearing fault diagnosis method with an easy concept and a good diagnostic success rate.

## Figures and Tables

**Table 1:** SKF6205 ball bearing structure parameters.

Roller Diameter	Pitch Diameter	Roller Number	Contact Angle
7.9 mm	39 mm	9	0°

**Table 2:** N205 cylindrical bearing structure parameters.

Ball Diameter	Pitch Diameter	Ball Number	Contact Angle
7.5 mm	38.5 mm	12	0°

**Table 3:** Operation conditions of different data files. DP—defect position; DD—defect diameter; ML—motor load; HP—horsepower.

Data File	DP	DD (inch)	ML (HP)	Data File	DP	DD (inch)	ML (HP)
No. 105	IR	0.007	0	No. 199	OR	0.014	2
No. 119	RE	0.007	1	No. 188	RE	0.014	3
No. 132	OR	0.007	2	No. 209	OR	0.021	0
No. 108	IR	0.007	3	No. 223	IR	0.021	1
No. 169	IR	0.014	0	No. 236	RE	0.021	2
No. 186	RE	0.014	1	No. 237	OR	0.021	3

IR represents inner ring; RE represents rolling element; OR represents outer ring.

**Table 4:** Diagnosis results of different methods. AFW—adaptive frequency window; SCS—sparse coding shrinkage; FK—Fast Kurtogram.

Data File	AFW-SCS	AFW	SCS	FK	Data File	AFW-SCS	AFW	SCS	FK
No. 105	√	×	×	×	No. 199	×	×	×	×
No. 119	×	×	×	×	No. 188	√	×	×	×
No. 132	√	×	×	×	No. 209	√	√	√	√
No. 108	√	√	×	√	No. 223	√	×	×	×
No. 169	√	√	×	√	No. 236	√	√	×	×
No. 186	√	×	×	×	No. 237	√	√	√	√

√ represents success, × represents failure.
